# Evaluating Ocular Healthcare Accessibility and the Severity of Emergencies during Times of Crisis

**DOI:** 10.3390/jcm13195962

**Published:** 2024-10-07

**Authors:** Jonas Neubauer, Paul Richter, Lisa Strudel, Focke Ziemssen, Spyridon Dimopoulos

**Affiliations:** 1 Center of Ophthalmology, Eberhard-Karls University Tuebingen, Elfriede-Aulhorn-Straße 7, 72076 Tuebingen, Germany; 2 Center of Ophthalmology, University of Leipzig, Liebigstraße 12, 04103 Leipzig, Germany

**Keywords:** eye care, emergency, pandemic, healthcare utilization, healthcare accessibility, obstacles to healthcare accessibility

## Abstract

**Background/Objectives:** The COVID-19 pandemic has profoundly impacted healthcare systems worldwide, including the delivery of ophthalmic emergency services. This study examines the impact of the COVID-19 pandemic on the clinical presentation of emergencies and the accessibility of healthcare in ophthalmology. **Methods:** The study employed a single-center, consecutive case series design with historical controls to examine electronic health records over a 21-day period during the COVID-19 pandemic and a matched period from the preceding year. Records were analyzed for demographic variables, diagnosis, length of stay, travel distance, and referral status. The urgency of cases was evaluated by three independent graders using the BaSe SCOrE (BAsic SEverity Score for Common OculaR Emergencies). **Results:** A total of 1229 patients were included in the study, with 786 patients in the 2019 cohort and 443 patients in the 2020 cohort. During the pandemic period, there was a significant decrease in the number of patients and the duration of their visits (*p* < 0.0001, *p* < 0.0001, respectively). There was an increase in walk-in patients (*p* = 0.03), who took significantly longer journeys to be treated as compared to referred patients (*p* < 0.01). At the same time, the severity of emergencies increased (*p* = 0.02). The 2019 logistic regression model found that age (*p* = 0.003), referral status (*p* < 0.001), distance (*p* = 0.009), and first presentation (*p* = 0.02) were significant predictors of the severity, while gender was not (*p* = 0.78). The 2020 model found that only age (*p* < 0.001) and referral status (*p* < 0.001) were significant predictors of severity. **Conclusions:** The observed decline in patient volume, increased severity of emergencies, and shifts in predictive variables within the logistic regression models are indicative of significant barriers to healthcare access. Therefore, enhancing health literacy and ensuring low-threshold access to emergency services are crucial, especially during crises.

## 1. Introduction

Effective healthcare systems are under constant tension due to changing demand and operational capacities, which becomes particularly evident in the events of crises. While localized hotspots often arise from conflicts or natural disasters, the coronavirus disease 2019 (COVID-19) pandemic was an unprecedented, global event in modern history [1]. In ophthalmology, it is crucial to differentiate between urgent and non-urgent diseases, as timely intervention can preserve the vision and health of patients [2]. A functioning ophthalmological emergency system has to adapt to changes in demand by efficiently allocating limited resources to manage all acute emergencies in a timely manner. These difficulties were highlighted by the emergence of the COVID-19 pandemic, with elective procedures being postponed in order to maintain capacities for more urgent emergencies. 

After the first COVID-19 cases in Europe were diagnosed in late January 2020 [3,4], restrictions on public life were imposed, including the closure of schools, universities, non-essential public services, and shops. While some countries introduced national curfews, people in Germany were always allowed to move freely inside the country [5]. Hospitals implemented guidelines to reduce the risk of COVID-19 infections [6,7]. With regards to ophthalmology, the discussions initially centered on the particularly high risk of COVID-19 transmission, considering the close contact during slit lamp examinations [8]. Although life-threatening diseases are rare in ophthalmology, it is important to examine the impact of the pandemic on ocular emergencies of varying urgency, especially as it is expected that patients change their behavior during times of stress, which may delay their presentation to emergency services and possibly lead to more severe diseases. Furthermore, the pandemic was demonstrated to have a negative effect on the accessibility of healthcare for patients on a global scale [9,10]. However, limited data is available concerning this influence specifically in the field of ophthalmology in Germany. 

Therefore, the aim of this study is to investigate the changes in patient demographics, the pattern and severity of ophthalmologic emergencies, and healthcare accessibility during the pandemic, compared to the pre-pandemic period. 

## 2. Materials and Methods

### 2.1. Study Design and Patients

This is a retrospective, single-center, consecutive case series study with historical controls. The electronic patient records of all patients presenting to the University Eye Hospital Tübingen on an emergency basis were analyzed. All demographic and clinical data were documented in our database during the patients’ complete visit and retrospectively analyzed. A study period after the introduction of public life restrictions from 3/23/20 to 4/13/20, which is also a public holiday (Easter Monday), was used. The comparison period from 2019 was chosen to reflect a comparable period of the previous year (4/1/19 to 4/22/19).

### 2.2. Ethical Considerations

This study adhered to the tenets of the Declaration of Helsinki and was approved by the local institutional ethics commission of the Eberhard Karls University, Tuebingen, Germany (2562021B02). Patient consent was waived due to the retrospective nature of the study and the pseudonymization of the clinical data.

### 2.3. Data Analysis

Pseudonymization was used for data analysis. The emergencies’ severity was classified with the BAsic SEverity Score for Common OculaR Emergencies [11]. The severity score of pathologies was assessed by three masked specialists independently of each other on the basis of the documented findings. In cases of disagreement, or if the diagnosis had not been described in the consensus, the median was then chosen to be used in the analysis. The distance from the patients’ address to the tertiary center was calculated using a commercial route planner [4]. Only patients living within 200 km of the center were included in this analysis. The distance traveled to the hospital was used as a quantifiable marker of healthcare accessibility. For comparison of the two study periods, a Chi-squared test or Fisher’s exact test was performed. If not otherwise indicated, the non-parametric statistical test (Wilcoxon signed-rank test) was performed. To assess the relationship between patients’ data and urgency of the condition in a logistic regression model, we grouped the patients into low (Base Score 0–2) and high urgency (Base Score 3–5) patients. For all statistical tests, a *p*-value < 0.05 was considered significant. Statistical analysis, logistic regression models (glm function of the base R package), and Figures were created with R (3.6.0).

## 3. Results

During the corresponding recording periods, only 56% of cases (443 vs. 786) were assessed during the pandemic (Table 1). The basic characteristics of the study groups showed a similar age of presenting patients. During the pandemic period, not only did the number of patients presenting to the tertiary center significantly decrease (*p* < 0.001), but there was also a substantial reduction in the duration of their stay (including waiting time). The median time from arrival to completion of treatment decreased significantly from 104 min pre-pandemic to 63 min in the pandemic scenario (*p* < 0.0001). The proportion of patients presenting to the clinic for the first time was comparable between the two periods under study (*p* = 0.9, Fisher’s exact test).

Table 2 illustrates selected diagnoses and the distribution of their frequency. In 2020, there were fewer contact lens associated keratitis and bacterial conjunctivitis, but more cases of foreign bodies (*p* < 0.0001, *p* = 0.03, *p* < 0.0001, respectively). Additionally, there was no significant increase in the incidence of retinal detachment or in the rate of macular-off presentations (*p* = 0.89 and *p* = 0.79, respectively).

**Figure 1:**
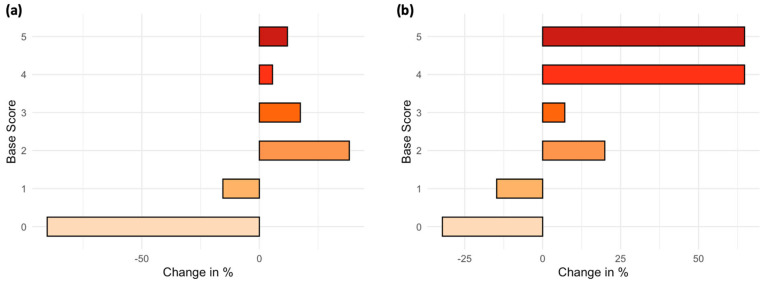
The change in Base Score between the pandemic and pre-pandemic period showed significant differences (*p* = 0.02). (**a**) Only for referred patients. (**b**) Only for self-referred patients.

The distribution of Base Scores showed a significant difference between the two study periods, with an increase in more urgent emergencies during the pandemic (Base Score ≥ 2) (*p* = 0.02) (Figure 1, Table 1).

**Figure 2:**
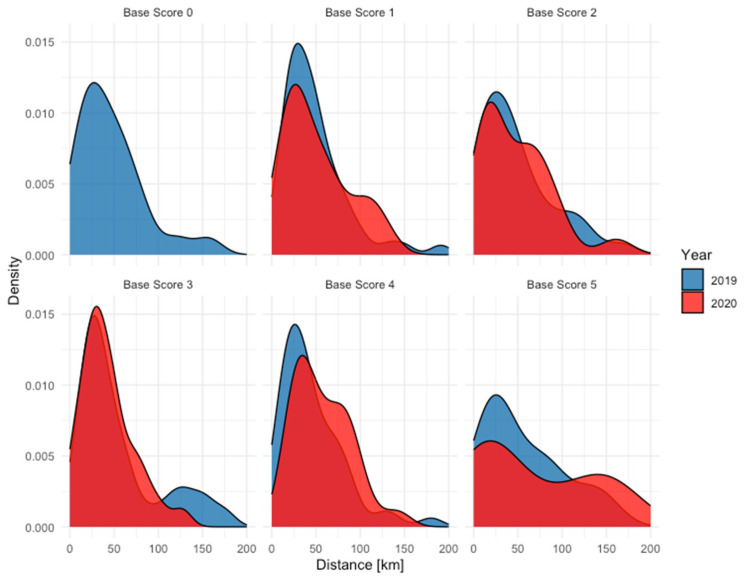
Distribution of the distance traveled by referred patients to reach the ophthalmology department divided by the severity of the emergency.

**Figure 3:**
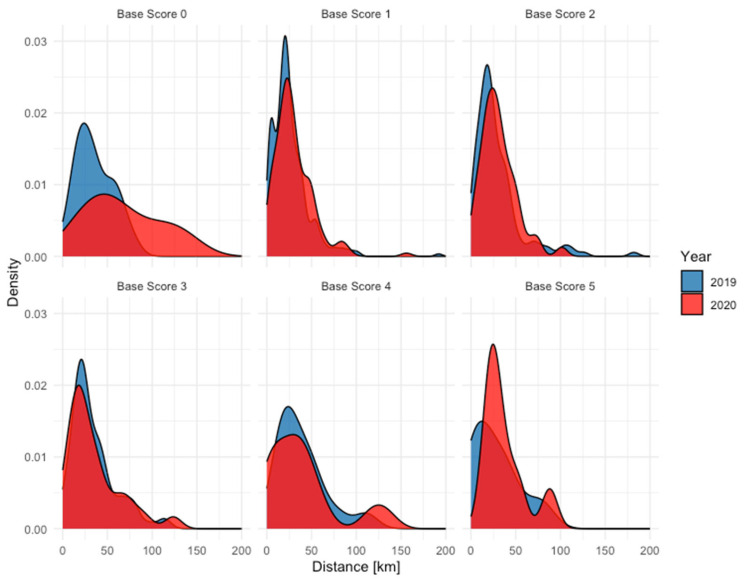
Distribution of the distance traveled by self-referred patients to reach the ophthalmology department divided by the severity of the emergency.

When analyzing the travel distance, a distinction was made between walk-in patients and referred patients. For referred patients, there was no significant difference in the distance traveled between the years 2019 and 2020, with mean distances of 49.8 km and 50.7 km, respectively. The distribution of these travel distances is detailed in Figure 2, which illustrates the consistency in travel patterns for referred patients over the two years. In contrast, walk-in patients showed a significant increase in the distance traveled in 2020 compared to 2019 (*p* < 0.01), with the mean distances rising from 28.8 km to 32.6 km. Figure 3 presents the distribution of travel distances for walk-in patients, highlighting the shift towards longer travel distances in 2020, visible especially for patients with a Base Score of zero.

The logistic regression models indicated several significant predictors to distinguish between low (Base Score 0–2) or high (Base Score 3–5) severity emergencies. The logistic regression model of 2019 indicated that age (β = 0.012, SE = 0.0042, *p* < 0.01), referral status (β = 1.45, SE = 0.18, *p* < 0.001), distance (β = 3.63 × 10^−6^, SE = 1.40 × 10^−6^, *p* < 0.01), and first presentation (β = −0.42, SE = 0.18, *p* = 0.02) were significant predictors, while gender was not (*p* = 0.78). A logistic regression analysis on the 2020 data revealed that age (β = 0.026, SE = 0.0054, *p* < 0.001) and referral status (β = 1.63, SE = 0.25, *p* < 0.001) were significant predictors of a severe emergency, while gender, distance, and first presentation were not significant predictors (*p* = 0.85, *p* = 0.33, *p* = 0.26, respectively).

## 4. Discussion

Although there was a slight shift in the degree of severity under reduced case numbers, this process confirmed the relevance of eyecare utilization. The significant decrease in absolute numbers and the increase in the macula-off status of detached retinas and travel distance to reach the clinic revealed the weaknesses of healthcare systems with low access barriers. Opportunistic screening or the recognition of emergencies, however, first and foremost requires the knowledge and awareness of those affected [12].

Although in the first few months of the pandemic there was great concern about overloading the emergency systems, the figures show that in the end it was more likely that delayed or non-existent emergency care was the result of crisis awareness. During the initial phase of the pandemic, there was a significant reduction in emergency consultations, with a 44% decrease in patient visits compared to pre-pandemic levels, which was consistent with findings from other countries [13,14].

While other ophthalmology departments described that patients stayed longer times in the clinic, we found that our patients spent significantly less time in the department despite the fact that patients during the pandemic presented with significantly more severe illnesses [15]. This underlines the effectiveness of the implemented changes and efficient patient flow.

Although the important quality indicators of emergency control were fulfilled, the implementation of admission controls, queues, and public scaremongering led to a notable reduction in the number of emergencies treated [16]. It is assumed that the actual incidence due to the pandemic had no influence on at least a large proportion of these cases.

However, a survey revealed that only 17% of patients with retinal detachment delayed their presentation due to the COVID-19 lockdown [17]. Other ocular diseases also showed changes associated with the endorsed social distancing and stay-at-home rules, such as lower rates of conjunctivitis and contact lens-related keratitis, as previously described [18,19,20]. In contrast, a surge of foreign bodies was observed as well, which could be explained by an increase in home improvement projects during stay-at-home orders [19].

Within our study cohort, we found an increase in patients with central artery occlusion or vein occlusion. Just as with COVID-19, these conditions are known to be associated with stroke or heart attack [21,22,23]. At the same time, studies have shown that across hospitals worldwide, admissions and treatments for strokes during the pandemic declined, indicating an unmet need for treatment of severe neurological and cardiovascular diseases [24,25]. However, larger retrospective studies did not show a significant increased risk for retinal vascular occlusion after a COVID-19 infection [26,27]. 

Even though emergencies in ophthalmology are mostly not life-threatening, some need urgent treatment to be resolved or to avoid further worsening resulting in permanent damage. Previous studies investigating the impact of the pandemic on ophthalmological emergencies have mainly focused on the reduced number of patient visits and operations [15,28]. However, the classification of the emergencies’ urgency was not carried out using a standardized and already verified system, but was often based on the assessment of individuals. When implementing a standardized classification protocol (Base Score), significant differences were identified: the frequency of less severe emergencies (Base Score 0–1) decreased, while the occurrence of severe cases increased. Similar changes have been reported in other studies [29,30]. In recent years, an increased number of self-referred patients presenting to emergency departments in Germany has been observed. This trend was not only evident in this sample, but across other specialties as well [31,32]. The rise seems to be mainly due to less urgent cases, with a survey showing that patients tend to overestimate their own urgency [33]. Therefore, looking only at self-referred patients, it was notable that we were able to identify a significant change in severity across the studied periods, with a decrease in low severity and an increase in medium and high severity cases. This raises the question as to whether patients with less urgent eye emergencies generally assess their situation more accurately than is currently believed. In addition, these changes could also be related to other influences such as access, the self-confidence of informed patients, and the freedom of choice of a healthcare system with compulsory health insurance. Further in-depth studies are needed to better understand this complex topic. 

While medical services remained generally available in Germany during the pandemic, closures of private practices and reduced public transport posed challenges for some patients, as has also been described in other countries [6]. To evaluate healthcare accessibility, we measured the distance patients had to travel to reach our clinic. Thereby we observed that referred patients traveled a similar distance to the eye clinic in comparison to the pre-pandemic controls. In contrast, self-referred patients demonstrated a significantly longer travel distance in order to get timely medical assistance, indicating obstacles to access ophthalmological healthcare. This can also be observed in the analysis of the two logistic regression models: while the distance traveled was a significant predictive factor for a severe emergency in 2019, the logistic regression model for 2020 no longer demonstrated any significant influence, meaning that patients with low-urgency emergencies also had to travel longer to receive qualified help. Furthermore, the logistic regression analysis from 2019 indicated that first presentation was a significant negative predictive factor for a serious emergency. However, this was no longer the case in 2020, which may reflect the overall increased severity of emergencies. Data on this subject within the field of ophthalmology are scarce. However, a study conducted in Israel demonstrated a significant increase of approximately 30% in the distance traveled during the pandemic [34]. These findings highlights the importance and potential of better education and healthcare accessibility for the general population to access emergency services in a timely manner, particularly during periods of crisis.

While our study gives valuable insights into the impact of the pandemic on emergency care in ophthalmology, it has limitations inherent to its retrospective and single-center design. Furthermore, we have considered the distance traveled as a quantifiable marker of healthcare accessibility, which does not account for several socio-economic variables (like organizational or financial accessibility) that also have an influence in this context. However, the presented data are particularly reliable due to Germany’s compulsory health insurance system, ensuring comprehensive coverage for all residents, and the fact that optometrists do not participate in emergency patient care within the German healthcare system [35,36]. It could therefore be assumed that the data presented poses a representative sample of all ophthalmologic emergencies. In addition, as country-specific lockdown laws have a strong influence on the populations’ healthcare accessibility, direct generalization of these results to other countries could be difficult.

In acute emergency care, there is a high need to care for patients according to their urgency and to prioritize or triage. For timely treatment, the initial delay before presentation to a specialist or tertiary care center is the first relevant hurdle and is of immense importance [37,38]. Here, the reliability of patient information, the possibility of self-triage, and the health literacy of affected patients have been proven to have room for improvement in the past [37]. Therefore, we believe that it is of the highest importance to educate the public about symptoms of urgent eye conditions, which should present as soon as possible to a specialized ophthalmology clinic. At the same time, the access to ophthalmologic emergency care should be simplified by improving the coverage of private practices open for emergency cases and by introducing a structured telemedicine framework for patients seeking urgent information [39]. In addition, studies utilizing support staff and algorithms have shown that they represent promising approaches to improve the efficiency of triage and care in ophthalmological emergencies [40,41].

## 5. Conclusions

The COVID-19 pandemic had a profound effect on the delivery of emergency care for patients in ophthalmology, revealing critical challenges to healthcare accessibility and utilization. Along with a reduced number of patient visits, an increase in the severity of ophthalmological emergencies was noticeable, suggesting delayed presentation. The observed increase in travel distances for self-referred patients further underscores the barriers to timely access, and emphasizes the urgent need for public education on eye health emergencies and enhanced accessibility to emergency ophthalmologic services, particularly during crises.

## Figures and Tables

**Table 1:** Basic characteristics of the study groups.

	Pre-Pandemic (2019)	Pandemic (2020)	*p*-Value
Total no. of patients	786	443	
Median no. of patients per day (min; max)	35 (21; 58)	19.5 (9; 40)	<0.0001 ^1^
Women, *n* (%)	381 (49)	200 (45)	0.24 ^2^
Median age (min; max) ≤6 7–65 >65	50 (0; 97) 38 553 195	48 (0; 93) 18 327 98	0.43 ^3^
Median time spent in the clinic (minutes)	104	63	<0.0001 ^1^
“Walk in” patients, *n* (%) Referred patients, *n* (%)	542 (69) 244 (31)	332 (75) 111 (25)	0.03 ^2^
Base Score, *n* (%) 0 1 2 3 4 5	40 (5) 339 (43) 170 (22) 141 (18) 68 (9) 28 (4)	8 (2) 171 (39) 121 (27) 85 (19) 40 (9) 18 (4)	0.02 ^3^
Diagnosis Category, *n* (%) Anterior Segment Glaucoma Neuroophthalmology Healthy Orbita/Oculoplastic Posterior Segment Uveitis	444 (56) 44 (6) 33 (4) 21 (3) 28 (4) 167 (21) 49 (6)	251 (57) 13 (3) 23 (5) 4 (1) 26 (6) 96 (22) 30 (7)	0.04 ^3^

^1^ Wilcoxon signed-rank test, ^2^ Fisher’s exact test, ^3^ Chi-square test.

**Table 2:** Incidence of selected diagnoses reported as numbers (%).

	Pre-Pandemic (2019)	Pandemic (2020)	*p*-Value ^1^
Conjunctivitis (bacterial)	73 (9.3)	14 (3.2)	<0.0001
Conjunctivitis (viral)	12 (1.5)	5 (1.1)	0.62
Keratitis (contact lens related)	9 (1.1)	- (-)	0.03
Keratitis (not contact lens related)	12 (1.5)	5 (1.1)	0.62
Central artery occlusion	2 (0.3)	4 (0.9)	0.20
Central vein occlusion	1 (0.1)	4 (0.9)	0.06
Retinal detachment Macular on Macular off	38 (4.8) 18 (47) 20 (52)	21 (4.7) 9 (43) 12 (57)	0.89
Retinal tear	14 (1.8)	7 (1.6)	1
Trauma	43 (5.5)	27 (6.1)	0.70
Orbital fracture	3 (0.4)	3 (0.7)	0.67
Foreign bodies	44 (5.6)	56 (12.6)	<0.0001
Chemical burn	3 (0.4)	1 (0.2)	1

^1^ Fisher’s exact test.

## Data Availability

The datasets presented in this article are not publicly available because of privacy reasons. Requests to access the datasets should be directed to the corresponding author.
